# Go and no-go learning in reward and punishment: Interactions between affect and effect

**DOI:** 10.1016/j.neuroimage.2012.04.024

**Published:** 2012-08-01

**Authors:** Marc Guitart-Masip, Quentin J.M. Huys, Lluis Fuentemilla, Peter Dayan, Emrah Duzel, Raymond J. Dolan

**Affiliations:** a Institute of Cognitive Neuroscience, University College London, London, W1CN 4AR, UK; b Wellcome Trust Centre for Neuroimaging, Institute of Neurology, University College London, London WC1N 3BG, UK; c Gatsby Computational Neuroscience Unit, University College London, London, W1CN 4AR, UK; d UCL Medical School, University College London, WC1N 4AR, UK; e Departament de Ciències Fisiològiques II, University of Barcelona, Institut d'Investigació Biomèdica de Bellvitge, 08907 L'Hospitalet de Llobregat (Barcelona), Spain; f Institute of Cognitive Neurology and Dementia Research, Otto-von-Guericke-University, Leipziger Strasse 44, 39120 Magdeburg, Germany

**Keywords:** Action, Learning, Pavlovian, Instrumental, Striatum

## Abstract

Decision-making invokes two fundamental axes of control: affect or valence, spanning reward and punishment, and effect or action, spanning invigoration and inhibition. We studied the acquisition of instrumental responding in healthy human volunteers in a task in which we orthogonalized action requirements and outcome valence. Subjects were much more successful in learning active choices in rewarded conditions, and passive choices in punished conditions. Using computational reinforcement-learning models, we teased apart contributions from putatively instrumental and Pavlovian components in the generation of the observed asymmetry during learning. Moreover, using model-based fMRI, we showed that BOLD signals in striatum and substantia nigra/ventral tegmental area (SN/VTA) correlated with instrumentally learnt action values, but with opposite signs for go and no-go choices. Finally, we showed that successful instrumental learning depends on engagement of bilateral inferior frontal gyrus. Our behavioral and computational data showed that instrumental learning is contingent on overcoming inherent and plastic Pavlovian biases, while our neuronal data showed this learning is linked to unique patterns of brain activity in regions implicated in action and inhibition respectively.

## Highlights

► Expectation of valence interferes with action learning in human participants. ► Computational modeling disentangles influences of instrumental and Pavlovian systems. ► Striatum and SN/VTA track action values and bind them to the control of vigor. ► Successful control is associated with activity in the inferior prefrontal cortex.

## Introduction

Optimal decision-making requires choices that maximize reward and minimize punishment. Animals are endowed with two broad classes of mechanisms to achieve this optimization. Firstly, hard-wired, or Pavlovian, policies directly tie affectively important outcomes, together with learned predictions of those outcomes, to valence-dependent stereotyped behavioral responses. Secondly, a more flexible, instrumental, controller learns choices on the basis of contingent consequences (Dickinson and Balleine, 2002). These controllers generally favor the same choices, thereby rendering learning fast and efficient. However, their underlying workings are best revealed by striking sub-optimalities that ensue when they come into opposition (Boureau and Dayan, 2011; Breland and Breland, 1961; Dayan et al., 2006).

One abundant source of sub-optimalities is the substantial interdependence of two logically independent axes of behavioral control (Boureau and Dayan, 2011; Cools et al., 2011; Gray and McNaughton, 2000; Niv et al., 2007): a valence axis running from reward to punishment, and an action axis running from vigor to inhibition. Pavlovian responses associated with predictions of reward usually entail vigorous active approach and engagement (Gray and McNaughton, 2000), irrespective of the instrumental validity of these actions. Equally, Pavlovian responses to (at least distal possible) punishments are generally associated with behavioral inhibition (Blanchard and Blanchard, 1988; Gray and McNaughton, 2000; Soubrie, 1986).

The functional architecture of the basal ganglia, a region known to support instrumental control, reflects the same association between affect and effect. For example, the so-called “direct pathway” promotes go choices in light of provided rewards while the “indirect pathway” promotes no-go choices in light of foregone rewards (Frank and Fossella, 2011; Gerfen, 1992). Further, the same dual association may also be expressed within ascending monoaminergic systems (Cools et al., 2011). Thus, the dopaminergic system is involved in generating active motivated behavior (Berridge and Robinson, 1998; Niv et al., 2007; Salamone et al., 2007) and instrumental learning through reward prediction errors (Schultz et al., 1997). On the other hand, the serotonergic system seems to be more closely affiliated with behavioral inhibition in aversive contexts (Crockett et al., 2008; Dayan and Huys, 2009; Soubrie, 1986).

Previous human studies on instrumental learning and decision making have generally exploited a conventional coupling between reward and go choices (e.g. Frank et al., 2004; O'Doherty et al., 2004). By contrast, various groups, including ourselves, have taken a different approach to decision-making, using tasks that fully orthogonalize action and valence in a balanced 2 (reward/punishment) × 2 (go/no-go) design (Crockett et al., 2009; Guitart-Masip et al., 2011). These latter tasks reveal that Pavlovian value expectations can disrupt instrumental performance, with anticipation of punishment impairing active go responses. However, the studies concerned considered steady-state behavior in a stable world, and did not examine learning. This is a critical omission, since the interaction between action and valence could boost, or indeed prevent learning altogether, and since the neural substrates of acquisition and maintenance could be quite different — as indeed has been claimed for action learning (Atallah et al., 2007; Everitt et al., 2008).

Here, we designed a variant of our previous task (Guitart-Masip et al., 2011) to examine Pavlovian influences on instrumental learning of go and no-go choices to maximize gains and minimize losses. This question has generally been studied using Pavlovian to instrumental transfer paradigms involving separate Pavlovian and instrumental training phases prior to a transfer phase in which the effects of Pavlovian stimuli on instrumental performance are tested in extinction (Cardinal et al., 2002; Huys et al., 2011; Parkinson et al., 1999; Talmi et al., 2008). Our task, instead, involves the instrumental learning of active and passive choices (go or no-go) in contexts where either wins or losses are probabilistically realized. Therefore, the expected Pavlovian effects are incidental. The task structure allows a detailed computational analysis of Pavlovian and instrumental influences during learning while retaining the orthogonalization of reward/punishment and go/no-go of our original task.

We hypothesized that learning of the optimal action choice (go or no-go) would be affected by the value of the choice outcomes. This would result from an interference arising out of state values or the expected value generated by the fractal images (Pavlovian controller) on the learned instrumental choice values for go and no-go options (Instrumental controller). In our task action and state values are indistinguishable from each other using fMRI because these values are highly correlated in some of the conditions. However, we envisaged that the neural correlates of action values for go and no-go choices would be affected by the states in which these actions are required. We expected that action values for go and no-go choices would be differentially expressed in the win and avoid losing conditions in brain areas implicated in the realization of a behavioral interaction between action and valence in our task. We surmised that this interaction should be evident in the striatum and amygdala, guided by previous studies implicating such regions in Pavlovian influences on instrumental choice (Cardinal et al., 2002; Parkinson et al., 1999; Talmi et al., 2008). Finally, as we also observed a value independent action bias in choices, we predicted that brain areas involved in inhibiting prepotent responses such as the inferior frontal gyrus (Aron and Poldrack, 2006; Robbins, 2007) would be involved in no-go performance.

In accordance with previous accounts of the involvement of the striatum and SN/VTA in instrumental learning, we show that the magnitude of activity in striatum and SN/VTA parametrically tracked instrumental action values. Critically, we show that the sign of relationship between action value and striatal and SN/VTA activity depended on the vigor status of the behavioral choice, being positive for go actions and negative for no-go actions. For instance, a larger expected reward for a no-go action was coupled to less activity in both striatum and SN/VTA, whereas a larger expected reward for a go action was coupled to more activity in the same structures. Moreover, we exploited the fact that a significant subset of participants did not acquire accurate instrumental responses for all conditions to characterize the differential neural responses in those participants that showed successful instrumental performance in our task.

## Materials and methods

### Subjects

47 adults participated in the experiment (28 females and 19 males; age range 18–35 years; mean 23.1, SD = 4.1 years). 17 subjects performed the experiment outside, and 30 subjects, inside the scanner. All participants were healthy, right-handed and had normal or corrected-to-normal visual acuity. None of the participants reported a history of neurological, psychiatric or any other current medical problems. All subjects provided written informed consent for the experiment, which was approved by the local ethics board (University College London, UK).

### Experimental design and task

**Fig. 1:**
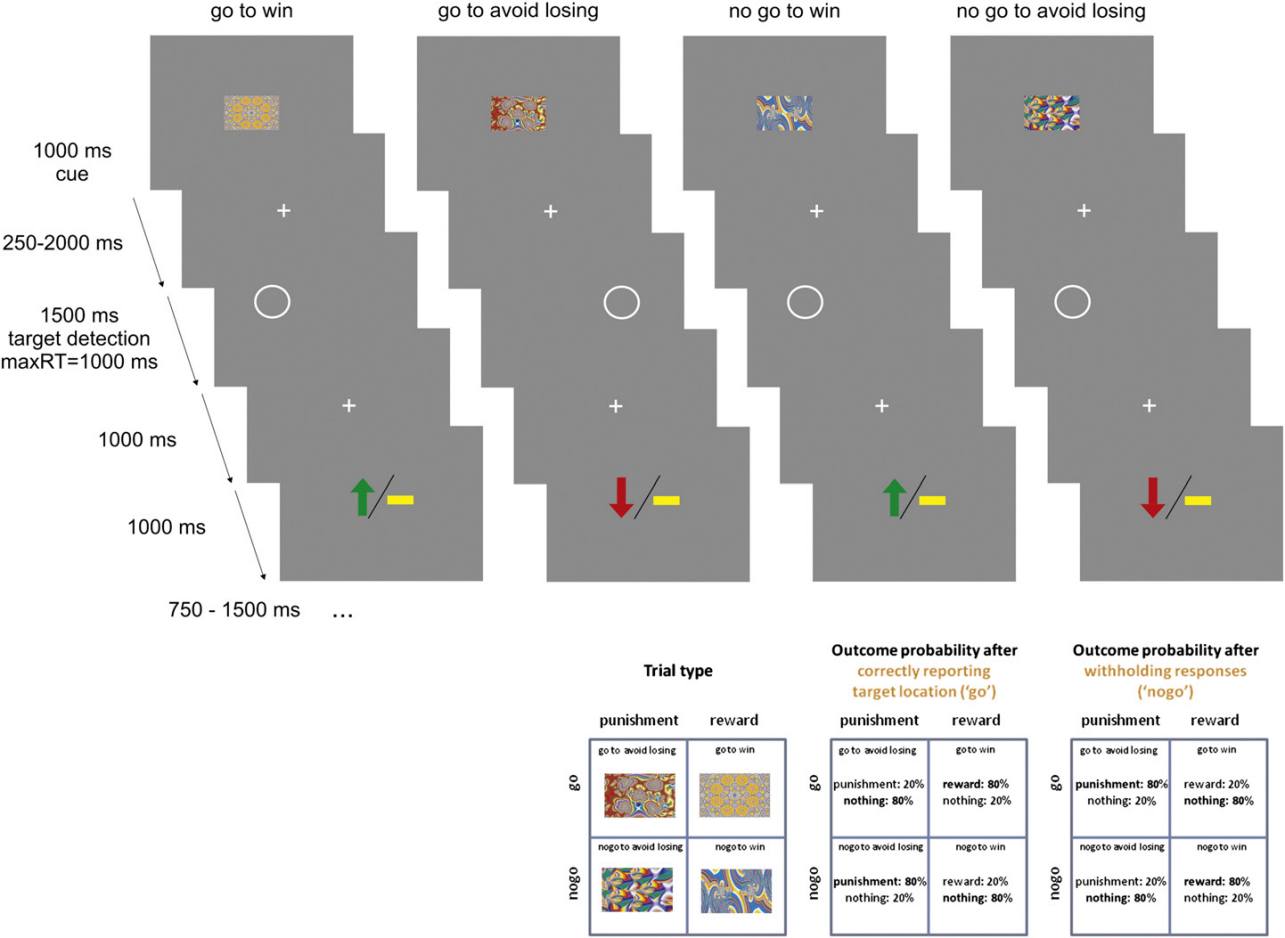
Experimental paradigm. On each trial one of four possible fractal images indicated the combination between action (making a button press in go trials or withholding a button press in no-go trials) and valence at outcome (win or lose). Actions were required in response to a circle that followed the fractal image after a variable delay. On go trials, subjects indicated via a button press on which side of the screen the circle appeared. On no-go trials they withheld a response. After a brief delay, the outcome was presented: a green upward arrow indicated a win of £1, and a red downward arrow a loss of £1. A horizontal bar indicated of the absence of a win or a loss. On go to win trials a correct button press was rewarded, on go to avoid losing trials a correct button press avoided punishment, in no-go to win trials a correct withholding a button press led to reward, and in no-go to avoid losing trials a correct withholding a button press avoided punishment. The schematics at the bottom represent for each trial type, the nomenclature to the left, the possible outcomes and their probability after a correct response to the target (go choice) in the middle, and the possible outcomes and their probability after withholding a response to the target (no-go choice) in the right.

We used a modified version of an experimental design we previously employed to disentangle the effects of action and valence in anticipatory responses in the striatum and the SN/VTA post learning (Guitart-Masip et al., 2011). Here we are addressing learning of state-action contingencies. Each trial consisted of three events: a fractal cue, a target detection task and a probabilistic outcome. The trial timeline is displayed in Fig. 1. In each trial, subjects saw one of four abstract fractal cues for 1000 ms. The fractal cues indicated whether a participant would subsequently be required to perform a target detection task by emitting a button press (go) or not (no-go). The fractal also instructed subjects as to the possible valence of any outcome consequent on the subject's behavior (reward/no reward or punishment/no punishment). The meaning of the fractal images was randomized across participants.

Following a variable interval (250–2000 ms) after offset of the fractal image, the target detection task commenced. The target was a circle displayed on one side of the screen for 1500 ms. Participants had 1000 ms in which they indicated, via a key press, the side on which the cue was presented. If they chose to do so, and if they chose the correct side, the response was classified as “go”. 1000 ms after the offset of the circle, subjects were presented with the outcome. The outcome remained on screen for 1000 ms: a green arrow pointing upwards indicated a win of £1, a red arrow pointing downwards indicated a loss of £1, and a yellow horizontal bar indicated no win or loss. The outcome was probabilistic, in win trials 80% of correct choices and 20% of incorrect choices were rewarded (the remaining 20% of correct and 80% of incorrect choices leading to no outcome), while in lose trials 80% of correct choices and 20% of incorrect choices avoided punishment.

Thus, there were 4 trial types depending on the nature of the fractal cue presented at the beginning of the trial: press the correct button in the target detection task to gain a reward (go to win); press the correct button in the target detection task to avoid punishment (go to avoid losing); do not press a button in the target detection task to gain a reward (no-go to win); do not press a button in the target detection task to avoid punishment (no-go to avoid losing). Unlike Guitart-Masip et al. (2011), in the current experiment, subjects were not verbally instructed about the action contingencies for each fractal image and had to learn them by trial and error. Participants were instructed that the correct choice for each fractal image could be either go or no-go. They were also instructed about the probabilistic nature of the task. Those participants that performed the task inside the scanner learned the task contingencies as they were being scanned.

Our task separated instrumental responses (go and no-go choices to the targets) from the fractal images that indicate action requirements and outcome valence in order to dissociate anticipatory brain responses from responses elicited by execution of an actual motor response. However, unlike our previous experiment (Guitart-Masip et al., 2011), in the current experiment all trials included both a target detection task and outcome delivery. This decreased power for detecting changes in BOLD responses uniquely associated with action anticipation, but ensured that the learning process was not confounded by any attempt to decorrelate these two factors. The anticipatory response of an action before actual execution of any motor component involves action invigoration and is likely to be associated with the deployment of cognitive resources (attention and sensory process) that allow a directing effect on the specific action being prepared. This dual association of motoric and cognitive components that interact to sculpt a motor response is a general mechanism that allows adaptive interactions with the environment. Assessing the extent to which invigoration of action, and the associated deployment of distinct cognitive resources, can be attributed specifically to the observed anticipatory responses in the midbrain/basal ganglia network goes beyond the immediate goals and scope of the present study.

The task included 240 trials, 60 trials per condition and was divided into four 9 min sessions (15 trials per condition). Subjects were told that they would be paid their earnings of the task up to a total of £35. Before starting with the learning task, subjects did 20 trials of the target detection task in order to get familiarized with the speed requirements.

### Behavioral data analysis

The behavioral data were analyzed using the statistics software SPSS, version 16.0. The number of correct choices in the target detection task (correct button press for go conditions and correct omission of responses in no-go trials) was collapsed across time bins of 10 trials per condition. These measures were analyzed with a three way repeated-measures ANOVA with time, action (go/no-go) and valence (win/lose) as factors. In an initial analysis we also included group (inside the scanner/outside the scanner) as a between-subject factor.

### Reinforcement learning models

We built six parameterized reinforcement learning models to fit to the behavior of the subjects. All the models assigned each action *a*_*t*_ on trial *t* a probability. This was based on an action weight *W*(*a*_*t*_, *s*_*t*_) that depended on the stimulus on that trial, and which was passed through a squashed softmax (Sutton and Barto, 1998):

(1) $$p\left(a_{t}|s_{t}\right)=\left[\frac{\exp(W\left(a_{t}|s_{t}\right)}{\sum_{a^{\prime }}\exp\left(W\left(a^{\prime }|s_{t}\right)\right)}\right]\left(1-\xi \right)+\frac{\xi }{2}$$

where *ξ* was the irreducible noise which was kept at 0 for one of the models (RW), but was free to vary between 0 and 1 for all other models.

The models further differed in terms of how the action weight was constructed. For models RW and RW + noise, *W*(*a*,*s*) = *Q*(*a*,*s*), which was a simple Rescorla–Wagner like update equation:

(2) $$Q_{t}\left(a_{t},s_{t}\right)=Q_{t-1}\left(a_{t},s_{t}\right)+e\left(\rho r_{t}-Q_{t-1}\left(a_{t},s_{t}\right)\right)$$

where *ε* was the learning rate. Reinforcements entered the equation through *r*_*t*_ ∈ {− 1, 0, 1} and *ρ* was a free parameter that determined the effective size of reinforcements for a subject. For model RW(rew/pun) + noise + bias, the parameter *ρ* could take on different values for the reward and punishment trials, but for all other models there was only one value of *ρ* per subject. This meant that those models assumed that loss of a reward was as aversive as obtaining a punishment.

The other models differed in the construction of the action weight in the following way. For model RW + noise + *Q*_0_, the initial *Q* value for the go action was a free parameter, while for all other models this was set to zero. For models that contained a bias parameter, the action weight was modified to include a static bias parameter *b*:

(3) $$W_{t}\left(a,s\right)=\{\begin{array}{l} Q_{t}\left(a,s\right)+b\;\text{if}\;a=\text{go}\\ Q_{t}\left(a,s\right)\;\text{else} \end{array}\text{.}$$

For the model including a Pavlovian factor (RW + noise + bias + Pav), the action weight consisted of three components:

(4) $$W_{t}\left(a,s\right)=\{\begin{array}{l} Q_{t}\left(a,s\right)+b+\pi V_{t}\left(s\right)\;\text{if}\;a=\text{go}\\ Q_{t}\left(a,s\right)\;\text{else} \end{array}$$

(5) $$V_{t}\left(s_{t}\right)=V_{t-1}\left(s_{t}\right)+e\left(\rho r_{t}-V_{t-1}\left(s_{t}\right)\right)$$

where *π* ≥ 0 was again a free parameter. Thus, for conditions in which feedback was in terms of punishments, the Pavlovian parameter inhibited the go tendency in proportion to the negative value *V*(*s*) of the stimulus, while it similarly promoted the tendency to go in conditions where feedback was in terms of rewards.

### Model fitting procedure

These procedures are identical to those used by Huys et al. (2011), but we repeat them here for completeness. For each subject, each model specified a vector of parameters **h**. We found the maximum a posteriori estimate of each parameter for each subject:

(6) $$h_{i}={\mathrm{argmax}}_{h}p\left(A_{i}|,h_{i}\right)p\left(h_{i}|\theta \right)$$

where *A*_*i*_ comprised all actions by the *i*th subject. We assumed that actions were independent (given the stimuli, which we omit for notational clarity), and thus *p*(*A*_*i*_|*h*_*i*_) factorized over trials, being a product of the probabilities in Eq. (1). The prior distribution over the parameters *p*(*h*_*i*_|*θ*) mainly served to regularize the inference and prevent parameters that were not well-constrained from taking on extreme values. We set the parameters of the (factorized) prior distribution *θ*, which consist of a prior mean *m* and variance *v*^2^, to the maximum likelihood given all the data by *all* the *N* subjects:

(7) $${\hat{\theta }}^{\mathrm{ML}}=\arg\max_{\theta }p\left(A|\theta \right)$$

(8) $$=\arg\max_{\theta }\left(\prod_{i}^{N}\int d^{N}h_{i}p\left(A_{i}|h_{i}\right)p\left(h_{i}|\theta \right)\right)$$

where *A* = {*A*_*i*_}_*i* = 1_^*N*^ comprised all the actions by all the *N* subjects and *θ* = {*m*, *v*^2^} were the prior mean and variance. This maximization was approximately achieved by Expectation–Maximization (MacKay, 2003). We used a Laplacian approximation for the E-step at the *k*th iteration:

(9) $$p\left(h|A_{i},\right)\gg N\left(h_{i}^{\left(k\right)},s_{i}^{\left(k\right)}\right)$$

(10) $$h_{i}^{\left(k\right)}=\arg\max_{h}p\left(A_{i}|,h\right)p\left(h|\theta^{\left(k-1\right)}\right)$$

where *N*(⋅) denotes a normal distribution and ∑ _*i*_^(*k*)^ is the second moment around *h*_*i*_^(*k*)^. This resulted in the following updates for the group-level parameters *θ* = {*m*, *v*^2^}:

(11) $$m^{\left(k\right)}=\frac{1}{N}\sum_{i}h_{i}^{\left(k\right)}$$

(12) $${\left(v^{\left(k\right)}\right)}^{2}=\frac{1}{N}\sum_{i}\left[{\left(h_{i}^{\left(k\right)}\right)}^{2}+S_{i}^{\left(k\right)}\right]-{\left(m^{\left(k\right)}\right)}^{2}\text{.}$$

Before inference, all parameters were suitably transformed to enforce constraints (log and inverse sigmoid transforms). All model fitting procedures were verified on surrogate data generated from a known decision process.

### Model comparison

Models would ideally be compared by computing the posterior log likelihood log*p*(*M*|*A*) of each model *M* given all the data *A*. As we had no prior on the models themselves (testing only models we believed were equally likely a priori), we instead examined the model log likelihood log*p*(*M*|*A*) directly. This quantity could be approximated in two steps. First, the integral over the hyperparameters was approximated using the Bayesian Information Criterion at the *group* level (Kass and Raftery, 1995):

(13) $$\log p\left(A|M\right)=\int d\theta p\left(A|\theta \right)p\left(\theta |M\right)$$

(14) $$\gg -\frac{1}{2}BIC_{\mathrm{int}}=\log p\left(A{|\hat{\theta }}^{\mathrm{ML}}\right)-\frac{1}{2}|M|\log\left(\left|A\right|\right)\text{.}$$

Importantly, however, $\log p\left(A|{\hat{\theta }}^{\mathrm{ML}}\right)$ was not the sum of individual likelihoods, but the integral over the individual parameters. We approximated this integral by sampling from the fitted priors:

(15) $$\log p\left(A|{\hat{\theta }}^{\mathrm{ML}}\right)=\sum_{i}\log\int dhp\left(A_{i}|,h\right)p\left(h|{\hat{\theta }}^{\mathrm{ML}}\right)$$

(16) $$\gg \sum_{i}\log\frac{i}{K}\sum_{k=1}^{K}p\left(A_{i}|h^{k}\right)$$

where *K* was set to 1000 and *h*^*k*^ were parameters drawn independently from the priors over the parameters $p\left(h|{\hat{\theta }}^{\mathrm{ML}}\right)$. These model comparison procedures were also verified on surrogate data generated from a known decision process. Comparing integrated BIC values is akin to a likelihood ratio test, and in fact can be shown to reduce to classical statistical tests for certain simple linear models (Kass and Raftery, 1995).

### fMRI data acquisition

fMRI was performed on a 3-Tesla Siemens Allegra magnetic resonance scanner (Siemens, Erlangen, Germany) with echo planar imaging (EPI). Functional data was acquired in four scanning sessions containing 135 volumes with 41 slices, covering a partial volume that included the striatum and the midbrain (matrix: 128 × 128; 40 oblique axial slices per volume angled at − 30° in the antero-posterior axis; spatial resolution: 1.5 × 1.5 × 1.5 mm; TR = 4100 ms; TE = 30 ms). This partial volume included the whole striatum, the SN/VTA, the amygdala, and the ventromedial prefrontal cortex. However, it excluded the medial cingulate cortex, the supplementary motor areas, the superior frontal gyrus, and the middle frontal gyrus. The fMRI acquisition protocol was optimized to reduce susceptibility-induced BOLD sensitivity losses in inferior frontal and temporal lobe regions (Weiskopf et al., 2006). Six additional volumes at the beginning of each series were acquired to allow for steady state magnetization and were subsequently discarded. Anatomical images of each subject's brain were collected using multi-echo 3D FLASH for mapping proton density (PD), T1 and magnetization transfer (MT) at 1 mm^3^ resolution (Weiskopf and Helms, 2008) and by T1 weighted inversion recovery prepared EPI (IR-EPI) sequences (spatial resolution: 1 × 1 × 1 mm). Additionally, individual field maps were recorded using a double echo FLASH sequence (matrix size = 64 × 64; 64 slices; spatial resolution = 3 × 3 × 3 mm; gap = 1 mm; short TE = 10 ms; long TE = 12.46 ms; TR = 1020 ms) for distortion correction of the acquired EPI images (Weiskopf et al., 2006). Using the FieldMap toolbox (Hutton et al., 2002) field maps were estimated from the phase difference between the images acquired at the short and long TE.

### fMRI data analysis

Data were analyzed using SPM8 (Wellcome Trust Centre for Neuroimaging, UCL, London). Pre-processing included realignment, unwrapping using individual fieldmaps, and spatial normalization to the Montreal Neurology Institute (MNI) space with spatial resolution after normalization of 1 × 1 × 1 mm. We used the unified segmentation algorithm available in SPM to perform normalization. This has been shown to achieve good intersubject co-registration for brain areas such as caudate, putamen and brain stem (Klein et al., 2009). Finally, data was smoothed with a 6 mm FWHM Gaussian kernel. The fMRI time series data were high-pass filtered (cutoff = 128 s) and whitened using an AR(1)-model. For each subject a statistical model was computed by applying a canonical hemodynamic response function (HRF) combined with time and dispersion derivatives (Friston et al., 1998).

Separate general linear models (GLMs) were fit to the data to address two distinct questions. First, we wanted to identify the neural underpinnings for the interaction between action and valence that we observed at the behavioral level. The computational model suggested this is related to an interaction between action and state values. Although the BOLD signal associated with these two values is indistinguishable in our paradigm, we hypothesized that an interference mediated by action and state values would be realized in an interaction between contextual valence (whether a trial had a positive or a negative state value) and action values for go and no-go choices. Therefore, our first GLM asked whether brain representations of instrumental values inferred from behavior, as per our best-fitting computational model, were dependent on the vigor status of the action (go versus no-go) and on the motivational setting (reward or punishment feedback). Second, we hypothesized that anticipatory responses to the fractal images would differ between those participants that successfully learned the experimental conditions and those that did not. Moreover, as we also observed a value independent action bias, we hypothesized that brain areas involved in inhibiting preponderant responses such as the inferior frontal gyrus (Aron and Poldrack, 2006; Robbins, 2007) would be involved in no-go performance. To address these questions, our second GLM was implemented to analyze the effects of action and valence anticipation (2 × 2 factorial design) during the anticipatory phase (fractal image), without using action values employed in the first GLM analysis.

#### GLM 1: effects of expected valence on the representation of action values (model-based analysis)

We built a general linear model that included 4 different conditions: 2 at the onset of the fractal images (anticipatory phase); and 2 at the onset of the outcome. At the onset of the fractal images, and at outcome onset, trials were divided into those with a positive expected value (go to win and no-go to win) and those with a negative expected value (go to avoid losing and no-go to avoid losing). The onset of fractal images was modeled using a boxcar that extended in time during the whole anticipatory phase until the target detection task was presented. Importantly, each of the onset regressors was parametrically modulated by two separate and independent regressors: one parametric regressor included the value of the go action (*Q*_*t*_(*go*)) and the other the value of the no-go action (*Q*_*t*_(*no-go*)). We modified the standard procedure implemented in SPM in order to prevent automatic orthogonalization of consecutive parametric regressors. These time-varying action values were updated according to Eq. (2) using the posterior learning rate for the winning model. This amounted to four parametric regressors in total for the anticipatory phase responses. During the outcome phase, each of the two conditions (positive and negative expected value conditions) was parametrically modulated by two independent regressors: one included the raw outcome value (0 or 1 for win trials; and 0 or − 1 in lose trials) and the other included the state value *V*_*t*_(*s*) as inferred by the model. Again, this resulted in a total of four parametric regressors for outcome phase responses. To capture residual movement-related artifacts, six covariates were included (the three rigid-body translations and three rotations resulting from realignment) as regressors of no interest. Two subjects had to be excluded from analysis because it was not possible to use their regressor for *Q*_*t*_(*no-go*) in the win trials as they did not make enough no-go choices to generate sufficient variance for the values to be used as a parametric modulator. Notice that these two participants show a selective poor performance for the no-go to win condition, as the performance in the other 3 conditions was higher than 80% in both cases.

To test for the effects of valence on different representations of action values, regionally specific condition effects were assessed by employing linear contrasts for each subject and each parametric condition (first-level analysis). The resulting contrast images were entered into a second-level random-effects analysis and the hemodynamic effects of each parametric condition were assessed using a 2 × 2 analysis of variance (ANOVA) with the factors ‘action’ (Q go/Q no-go), and valence (win/lose).

To test for the presence of reward prediction errors at the time of the outcome as well as effects of valence on outcome processing, regionally specific condition effects were tested by employing linear contrasts for each subject and each parametric condition (first-level analysis). The resulting contrast images were entered into a second-level random-effects analysis and the hemodynamic effects of each parametric condition were assessed using a one way analysis of variance (ANOVA) with four levels: raw outcome value in win trials, raw outcome value in lose trials, expected value in win trials, expected value in lose trials.

#### GLM 2: neural correlates of successful instrumental control

We built a second general linear model that included our 4 conditions of interest as separate regressors at the onset of the fractal images: go to win trials, go to avoid losing trials, no-go to win trials, and no-go to avoid losing trials. We also modeled the onset of the target detection task separately for trials in which subjects emitted (or did not emit) a button press. Note we intentionally included these two regressors in order to explain away variance associated with the performance of the motor response in the anticipatory phase responses. We also included, as a regressor, the onset of the outcome (which could again be win £1, lose £1, or no monetary consequence). To capture residual movement-related artifacts, six covariates were included (the three rigid-body translation and three rotations resulting from realignment) as regressors of no interest.

A heterogeneity in the expression of instrumental learning across subjects is well established (Schonberg et al., 2007). This was also the case here, with some subjects performing well in all conditions and others contributing the majority of errors. In fact this heterogeneity has advantages in that it allowed us to explore brain responses associated with appropriately successful instrumental control. To define successful instrumental control we used an arbitrary threshold of 60% correct trials across the whole experiment, and 80% correct in the second half of the experiment in every condition, enabling us to segregate subjects into learners (19/30) and non-learners (11/30). These criteria ensured participants classified as learners showed satisfactory instrumental learning in all four conditions (see supplemental Fig. S1). When we applied the same criteria to those participants that performed the task outside the scanner, we found that the proportion of learners was 7/17. A chi square test did not detect any differences in the frequency of learners between the two groups (*χ*^2^ = 2.16, ns).

We analyzed neural representations of valence (win/lose) and action (go/no-go) anticipation elicited by presentation of fractal images, independently from value representations. We focused on the time point at which the fractal stimuli were presented, prior to the presentation of the target that occasions a behavioral response. We first focused our analysis on the learners because they were likely to anticipate the correct action in all conditions, reflecting successful instrumental control. We then conducted a separate analysis comparing anticipatory responses between learners and non-learners to detect whether the pattern of activated areas found in the learners was specific to those subjects showing successful instrumental control.

To test for the effects of action and valence anticipation in learners, we tested for regionally specific condition effects in linear contrasts for each subject and each condition (first-level analysis). The resulting contrast images were entered into a second-level random-effects analysis and the hemodynamic effects of each condition were assessed using a 2 × 2 analysis of variance (ANOVA) with the factors ‘action’ (go/no-go), and valence (win/lose). To test for differences in the effects of action and valence anticipation between learners and non-learners we computed, at the first level, the parameter estimate of the main effect of action contrast [(go to win + go to avoid losing) − (no-go to win + no-go to avoid losing)] and the main effect of valence contrast [(go to win + no-go to win) − (go to avoid losing + no-go to avoid losing)]. The resulting contrast images were entered into a second-level random-effects analysis and the differences between the two groups (learners and non-learners) were assessed using a two sample *t*-test.

### Regions of interest

Predicted activations detected in our voxel-based analysis were corrected for multiple comparisons using small volume correction (SVC) within anatomically defined regions of interest: these comprised the striatum, the inferior frontal gyrus (IFG), and the substantia nigra/ventral tegmental area (SN/VTA) of the midbrain (main origin of dopaminergic projections). A priori, we also included the amygdala but as we did not observe any active voxel there, this ROI is not reported any further. The striatum and the IFG regions of interest (ROIs) were defined using the MNI templates available in Marsbar (Brett et al., 2002); the striatum ROI included the caudate and the putamen, whereas the IFG ROI included the pars trigeminalis and the pars opercularis of the inferior frontal gyrus. The SN/VTA ROI was manually defined, using the software MRIcro and the mean MT image for the group. On MT-images the SN/VTA can be distinguished from surrounding structures as a bright stripe (Bunzeck and Duzel, 2006). It should be noted that in primates, reward responsive dopaminergic neurons are distributed across the SN/VTA complex and it is therefore appropriate to consider the activation of the entire SN/VTA complex rather than, a priori, focusing on its subcompartments such as the VTA (Duzel et al., 2009). For this purpose, a resolution of 1.5 mm^3^, as used in the present experiment, allowed a sampling over 200 voxels of the SN/VTA complex, which has a volume of 350 to 400 mm^3^.

## Results

### Reward and punishment differently affects go and no-go choices

**Fig. 2:**
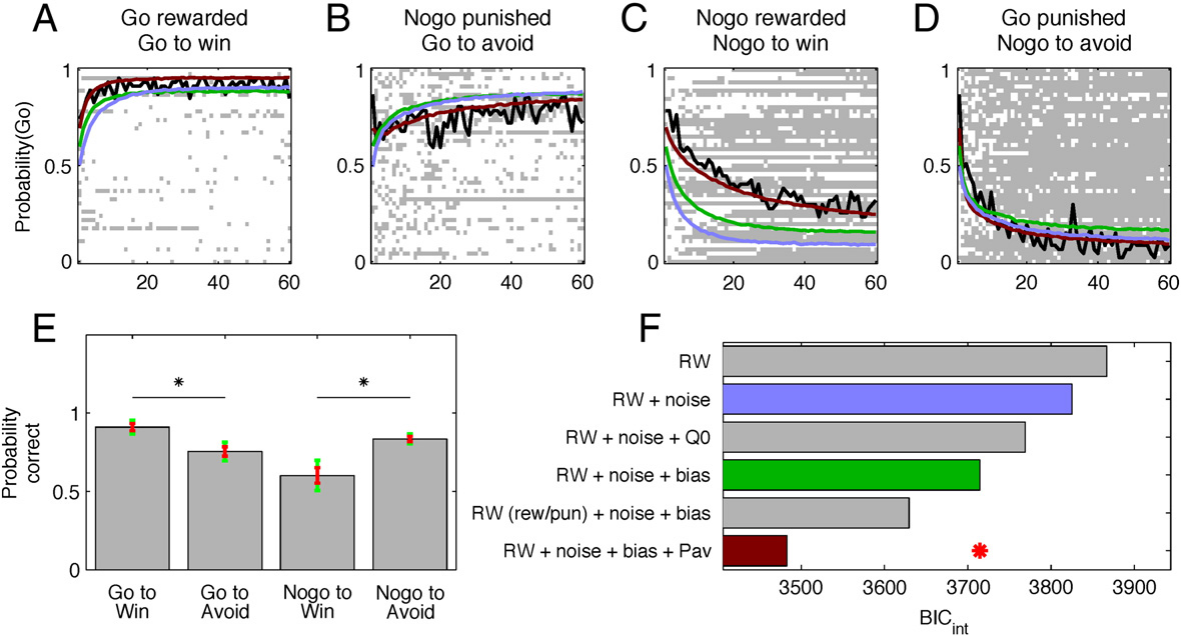
Observed and modeled behavioral performance. (A–D) Learning time courses for all four conditions. Each row of the raster images shows the choices of one of the 47 subjects in each of the four conditions. Go responses are depicted in white and no-go responses are depicted in grey. The overlaid black lines depict the time varying probabilities, across subjects, of making a go response. The colored lines show the same time-varying probabilities, but evaluated on choices sampled from the model (see Materials and methods). (E) Mean percentage of correct responses in each of the four conditions. Green error bars depict the 95% confidence interval (CI) and the red error bars depict standard error of the mean (SEM). Post hoc comparisons were implemented by means of repeated measures *t*-test: *p < 0.005. (F) Integrated Bayesian Information Criterion (BIC) score for all models tested. All models are modified Q-learning model with two pairs of action-values (go and no-go) for each state (fractal image). The winning model includes as free parameters a learning rate, a slope of the softmax rule, irreducible noise, a constant bias factor added to the action-value for go, and a Pavlovian factor that adds a fraction of the current state value to the action-value for go.

The optimal choice on both “go to win” and “go to avoid losing” trials is to go. Conversely, the optimal choice is not to emit an action in “no-go to win” and “no-go to avoid losing” trials. Figs. 2A–D show raw and average choice probabilities for all subjects. The group learning curves for each of the four conditions show that subjects did learn in all four conditions, but learning was far from equivalent across trial types. A three way ANOVA on the number of correct (optimal) choices with factors time (6 time bins of 10 trials each), action (go/no-go) and valence (win/lose) as repeated factors revealed a main effect of time (F(5,225) = 31.16, p < 0.001), a main effect of action (F(1,45) = 14.51, p < 0.001), and an action by valence interaction (F(1,45) = 40.15, p < 0.001), but no main effect of valence (F(1,45) = 1.64, p = 0.21). Note that overall, the percentage of trials in which subjects responded incorrectly in the target detection task (that is left when the target was on the right, or alternatively right when the target was on the left) was less than 0.1%. This result shows that participants had no problem solving the detection task accurately and, most importantly, that the effects of action and valence in our task cannot be explained by incorrect target detection task performance.

Thus, our raw behavioral data indicate that while subjects were equally good at learning from rewards and punishments, they showed better performance in conditions requiring a go choice than in trials requiring a no-go choice. Importantly, participants were better at learning to go in the reward condition (compared to go in the punishment condition), and were better at learning to withhold a response (no-go) in the punishment condition (compared to a similar response in the reward condition). This pattern was also evident in the total number of correct choices as a function of each condition (Fig. 2E, post-hoc paired *t*-tests, *t*(46) = 4.85, p < 0.001 and *t*(46) = 5.08, p < 0.001, respectively). Combined, these results constitute evidence for a striking interdependence of action and valence where rewards preferentially support learning of active go choices, and punishments preferentially support learning of no-go choices. These results cannot be accounted for by the asymmetries among trial types inherent to our experimental design, because the interaction that we found between action and valence is orthogonal to them. Whereas go and no-go conditions differ in the levels of cognitive effort because the go choices require a target detection task that is irrelevant for no-go choices, this difference cannot explain why participants reverse the level of accurate choices for go and no-go conditions depending on the valence of the outcomes. Similarly, whereas the objective expected value of a correct choice is lower in the avoid losing conditions, this difference cannot account for different effects of this difference on go and no-go choices.

There was no interaction between the two groups of subjects performing the task inside and outside the scanner: group by action (F(5,45) = 1.23; p = 0.28), group by valence (F(1,45) = 0.41; p = 0.56), group by time (F(5,225) = 1.23; p = 0.3), or group by action by valence interaction (F(1,45) = 1.42; p = 0.22). Consequently, the two groups were pooled in the computational analyses detailed below. By pooling the data from both behavioral experiments we increased the power of the expectation maximization-based fitting procedure (see Materials and methods for details).

### Action bias, instrumental learning, and Pavlovian responses compete for behavioral control

Reinforcement learning (RL) models can parameterize a fine-grained account of the interaction between action and valence as agents learn the reward structure of the environment. We adapted the parameters of a nested collection of models incorporating different instrumental and Pavlovian RL hypotheses to the observed behavioral data, and compared the parsimony and fit of their explanations using an integrated BIC (Bayesian Information Criterion) that takes an appropriately conservative account of the number of parameters used (see Materials and methods for details). Since we sought group inferences, and used the same model for all subjects in the imaging analyses, we compared the models at the group level, penalizing complexity after integrating out (by sampling) individual model parameters (see Materials and methods). We also generated surrogate data from the models by using the subjects' a posteriori parameters and simulating behavior in the task.

The base model was purely instrumental Q-learning devoid of any valence interaction (*Model RW* in Fig. 2F). This used a Rescorla–Wagner update rule to track the action value of each choice given each fractal image (*Q*_*t*_(*go*) and *Q*_*t*_(*no-go*)) independently. On each trial *t*, the model only updated the value of the action chosen on that trial. The probability of choosing the go action on trial *t* was a sigmoid function of the difference between the action values scaled by a slope parameter.

Even in the best condition (i.e. go to win; see Fig. 2A), subjects continued to make errors (albeit at a low rate) after reaching an early asymptote. We found that augmenting model RW with an irreducible action noise (*Model RW* + *noise* in Fig. 2F; see Talmi et al., 2009) improved our measure of parsimony BIC_int_ (integrated Bayes information criteria) (Fig. 2F). Surrogate data generated from RW + noise are shown in light blue in Figs. 2A–D.

However, these models still failed to capture an initial bias that subjects invariably exhibited toward performing the target detection task (in Figs. 2A–D, the initial probability of a go choice was always > 0.5). Consequently, we tested two alternative models to account for this effect: firstly, we included an initial shaping bonus (Ng et al., 1999) that could be naturally erased as the subjects learned (*Model RW* + *noise* + *Q*_0_ in Fig. 2F); or, secondly, we included a bias that was constant across the experiment (*Model RW* + *noise* + *bias* in Fig. 2F). The BIC measure favored the latter. Indeed the model's simulated behavior matched the true behavior better, particularly in the early stages (green lines in Figs. 2A–D).

However, the model *RW* + *noise* + *bias* still failed to capture the crucial action by valence interaction, as is clearly evident in the figures, for example in the no-go to win condition. Thus, we tested a further model that added a Pavlovian approach/withdrawal component to the other, instrumental, components. In this model, the probability of a go action was incremented proportionally to the overall (action-independent) state value of each stimulus. This model assumed that increasing reward expectancy induced a parametric increase in go probability, and that increasing punishment expectancy induced a parametric increase in no-go probability. For example, consider the no-go to win condition: as subjects learned to withhold their responses during the task, the stimulus indicative of this condition came to be associated with more reward. This positive expectancy, in turn, promoted a (inappropriate) go action. Similarly, the stimulus indicating the go to avoid losing condition embodied a negative expectation (even when a subject was always right, due to probabilistic feedback). In the model, this negative expectancy promoted inhibition of the requisite go choice. In both cases, we hypothesized that this Pavlovian factor would account for the pattern of action/valence interactions we observed, since on the one hand it should produce the very interference with performance in those critical conditions where action and valence were not aligned, while on the other hand it should support behavior in those conditions where action and valence were aligned.

Indeed, we found that this latter model (*Model RW* + *noise* + *bias* + *Pav* in Fig. 2F) provided the most parsimonious account of our data. Surrogate choices generated from the model showed that it accurately captured crucial differences in learning across conditions. This model predicted the choices of 43/47 subjects better than chance (binomial test, p < .05, geometric mean predictive probability 8 × 10^− 11^). Finally, we verified that the asymmetry was not due to differences in reward and punishment sensitivity by assessing a model with separate parameters for each (*Model RW*(*rew*/*pun*) + *noise* + *bias* in Fig. 2F).

Thus, our computational analysis strongly suggested that *three* factors contributed to the control of choice behavior during action-valence learning. First, we found evidence for associative reinforcement learning (the RW component), which we refer to as an Instrumental controller. Second, we found evidence for a strong and persistent bias toward emitting a go choice. Lastly, we found evidence for a Pavlovian coupling between action and valence expectation.

### Instrumental action values are represented in the striatum and the SN/VTA

**Fig. 3:**
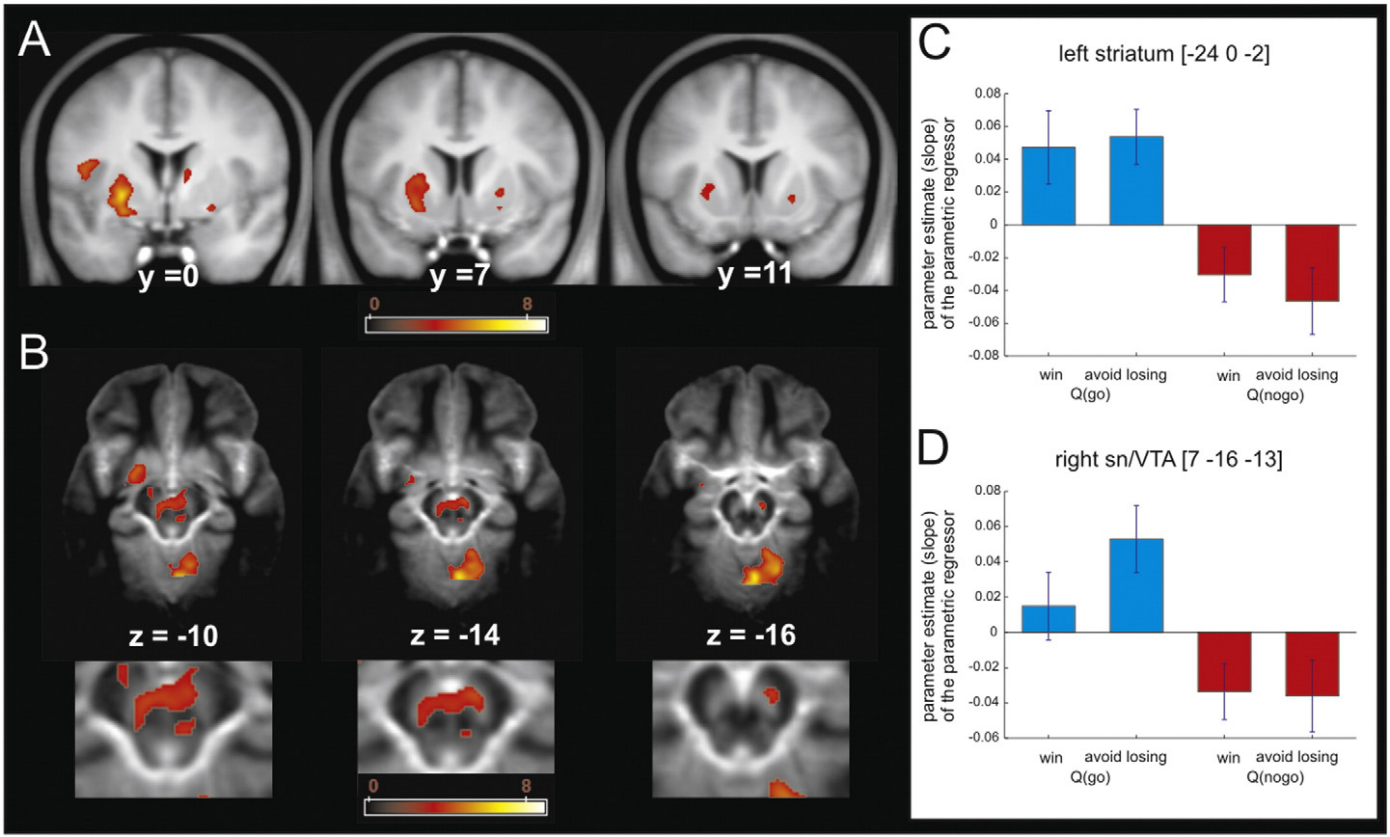
Action value representation in the striatum and SN/VTA. (A–B) The striatum (A) and the SN/VTA (B) show higher representation of Qgo when compared to Qno-go (p < 0.001 uncorrected; p < 0.05 SVC). The color scale indicates T values. (C–D) Parameter estimates of the four parametric regressors at the peak coordinate in the left putamen (C) and SN/VTA (D) showing that BOLD signal increased as the value of the go choice (Q go) increased both in the win and lose trials. On the other hand, BOLD signal decreased as the value of the no-go choice (Q no-go) increased, (note these parameter estimates were not used for statistical inference).

It would be conventional to have found a correlation between the fMRI BOLD signal in areas involved in decision-making, such as the striatum, and the values or propensities for choices, such as *Q*_*t*_(*go*) and *Q*_*t*_(*no-go*). However, the strong Pavlovian effects observed in the model suggested an effect of valence (winning versus avoiding losing) on the coding of action values or, concomitantly, an effect of action (go versus no-go) on the coding of predictions of valence. To assess the former, we created separate parametric regressors associated with *Q*_*t*_(*go*) in the two valence conditions, and separate parametric regressors associated with *Q*_*t*_(*no-go*) in the same two valence conditions. We then performed a voxel based 2 × 2 ANOVA on the regression coefficients. This showed a significant main effect of action (go versus no-go; see Fig. 3) in the left ventral putamen [Montreal Neurological Institute (MNI) space coordinates (x,y,z) − 24,0,−2; peak Z score = 5.61; p = 0.001 FWE], and bilateral SN/VTA [MNI space coordinates (x,y,z) 7,−16,−13; peak Z score = 3.96; p = 0.005 FWE SVC; MNI space coordinates (x,y,z) − 6,−18,−12; peak Z score = 3.78; p = 0.01 FWE SVC]. Note that this main effect of action is different from a simple comparison between trials requiring go and no-go choices. Instead, this main effect of action involves a comparison between separate action values for go and no-go choices on every trial.

A main effect of action implies that there was a significant difference between the regression coefficients associated with *Q*_*t*_(*go*) and *Q*_*t*_(*no-go*), but the test is agnostic as to the sign of the correlation between BOLD signal and the parametric regressors. Strikingly SPM parameter estimates in the activated clusters revealed that, throughout the previously highlighted brain areas, action values for the go choices (*Q*_*t*_(*go*)) were positively related to BOLD responses, while action values of a no-go choice (*Q*_*t*_(*no-go*)) were negatively related with BOLD responses. This differential implementation of go and no-go actions is evidence that a system involving the striatum and SN/VTA mediates behavioral control via a bi-directional binding of action value and vigor.

Neither of the other two facets of the 2 × 2 ANOVA was significant, i.e., there was neither a main effect of valence nor an action by valence interaction. Given the tight coupling between action values and Pavlovian values in our task (but see below for additional analysis of this issue), the lack of a main effect of valence suggested that we failed to find a locus coding for valence that could realize the Pavlovian effects revealed by our computational model. Future refinement in experimental design might enable detection of just such a segregated system, as indeed has been previously observed (Balleine et al., 2009), including in our laboratories (Dayan and Daw, 2008; Guitart-Masip et al., 2010; Talmi et al., 2008). A final point worthy of note is that we did not find any brain region coding for action values independent of action.

For completeness, we also report the responses to the outcome although we do not discuss them in detail. A brain area reporting reward prediction errors should have positively correlated with the raw outcome value and simultaneously negatively correlated with expected value. We did not find any brain area in which BOLD responses correlated (positively or negatively) with expected value at the time of the outcome. On the other hand, BOLD responses positively correlated with the raw outcome both in the win and the lose trials in the ventral striatum including the nucleus accumbens [MNI space coordinates (x,y,z) 12,6,−11; peak Z score > 7; p < 0.001 FWE] and the ventromedial prefrontal cortex [MNI space coordinates (x,y,z) − 7,45,−13; peak Z score = 7.14; p < 0.001 FWE]. This result suggested that these brain areas responded when an outcome was better than expected, that is a win in the win trials and the avoidance of a loss in the losing trials.

### Recruitment of SN/VTA and IFG is related to successful instrumental control

**Fig. 4:**
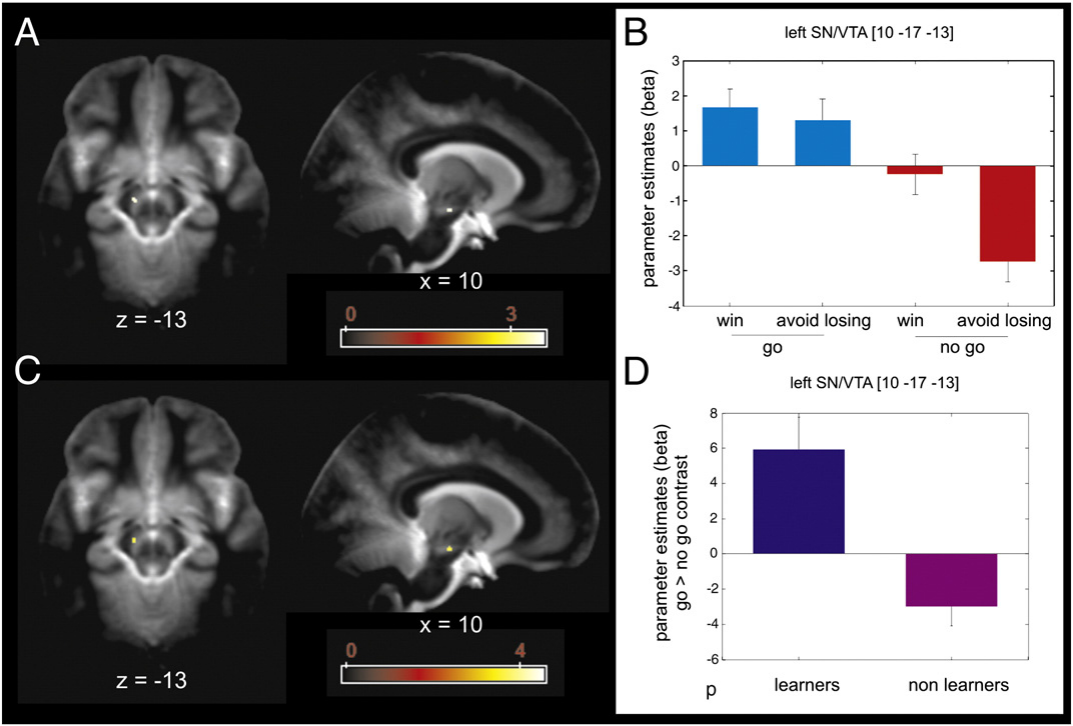
Action anticipation in learners and comparison to non-learners. (A) In learners, stimuli indicating go trials elicited greater left lateral substantia nigra/ventral tegmental area (SN/VTA) activity than stimuli indicating no-go trials (p < 0.001 uncorrected; p < 0.05 SVC). The color scale indicates T values. (B) Parameter estimates at the peak coordinates in the left lateral SN/VTA show activation at this location signals anticipation of action regardless of outcome valence (reward or punishment avoidance). Coordinates are given in MNI space. Error bars indicate SEM (note that these parameter estimates were not used for statistical inference). (C) In an independent comparison, left lateral SN/VTA distinguishes learners from non-learners in the magnitude of the contrast *go versus no-go* (p < 0.001 uncorrected; p = 0.05 SVC). The color scale indicates T values. (D) Parameter estimates at the peak coordinates in the left lateral SN/VTA show that only in subjects that learned, the task fractal images indicating go trials elicited higher BOLD activity than fractal images indicating no-go trials. Coordinates are given in MNI space. Error bars indicate SEM (note that these parameter estimates were not used for statistical inference).

**Fig. 5:**
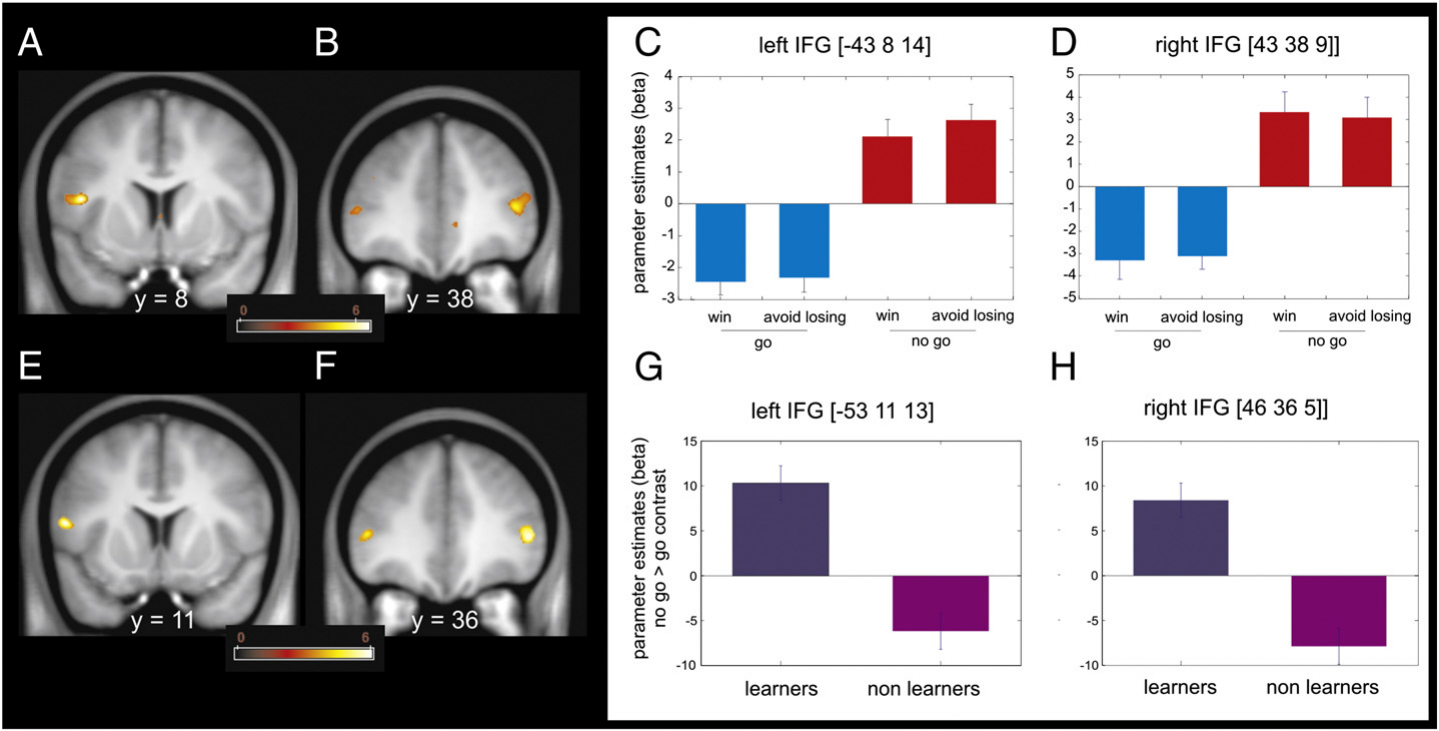
Inhibition anticipation in learners and comparison to non-learners. (A) In learners, stimuli indicating no-go trials elicited greater bilateral inferior frontal gyrus (IFG) activity than stimuli indicating go trials (p < 0.001 uncorrected; p < 0.05 SVC). The color scale indicates T values. (B) Parameter estimates at the peak coordinates in both IFG clusters show that activation at these locations signals a requirement for inhibition regardless of the trial outcome valence (reward or punishment avoidance). Coordinates are given in MNI space. Error bars indicate SEM (note that these parameter estimates were not used for statistical inference). (C) In an independent comparison, bilateral IFG distinguishes learners from non-learners in the magnitude of the contrast *no-go versus go* (p < 0.001 uncorrected, p < 0.05 SVC). The color scale indicates T values. (D) Parameter estimates at the peak coordinates in the clusters depicted in C show that only in subjects that learned the task, fractal images indicating no-go trials elicited higher BOLD activity than fractal images indicating go trials. Coordinates are given in MNI space. Error bars indicate SEM (note that these parameter estimates were not used for statistical inference).

Figs. 4A–B show a voxel based 2 × 2 ANOVA with factors of action (go and no-go) and valence (win and lose) restricted to the learners on the brain responses elicited by presentation of fractal images (anticipation) and independent from value representations. There was no main effect of valence or an action by valence interaction within the striatum, the SN/VTA, or the IFG. However, the analysis revealed a simple main effect of ‘action’ (go > no-go) in a sole cluster that survived SVC within our anatomical SN/VTA ROI located in left lateral SN/VTA [MNI space coordinates − 10,−17,−13; peak Z score = 3.38; p = 0.041 FWE SVC]. Furthermore, on the same analysis, subjects classified as learners revealed a complementary main effect of inaction (no-go > go) (Figs. 5A–B) in left IFG pars opercularis [MNI space coordinates − 43,8,14; peak Z score = 5.68; p < 0.001 FWE SVC], right IFG pars trigeminalis [MNI space coordinates 48,38,9; peak Z score = 4.66; p = 0.009 FWE SVC] and left IFG pars trigeminalis [MNI space coordinates − 45,31,4; peak Z score = 4.38; p = 0.027 FWE SVC].

Importantly, these patterns of BOLD responses differentiated learners from non-learners. A separate voxel based two-sample *t*-test involving a ‘go > no-go’ contrast between learners and non-learners also revealed a cluster of activation that survived SVC within our a priori SN/VTA ROI (Figs. 4C–D). This cluster located to the same coordinates (left lateral SN/VTA [MNI space coordinates − 10,−17,−13; peak Z score = 3.33; p = 0.054 FWE SVC]) and showed higher parameter estimates for the learners. Thus, remarkably, both analyses highlight the same peak voxel, suggesting that the left SN/VTA is specifically recruited in go trials for subjects who successfully learn and who (one assumes by learning) anticipate the appropriate choice (go or no-go) upon presentation of the relevant fractal images.

Finally, a separate voxel based two sample *t*-test comparing the magnitude of the ‘no-go > go’ contrast between learners and non-learners, we found three clusters of activation within an IFG anatomical ROI that survived SVC (Figs. 5C–D). These were located in close proximity to the foci of activation detected for the ‘no-go > go’ contrast in the learners: the left IFG pars opercularis [MNI space coordinates − 53,11,13; peak Z score = 4.77; p = 0.008 FWE SVC], the right IFG pars trigeminalis [MNI space coordinates 46,35,5; peak Z score = 4.68; p = 0.011 FWE SVC] and the left IFG pars trigeminalis [MNI space coordinates − 45,32,4; peak Z score = 4.26; p = 0.055 FWE SVC]. Note that the sign for the main effect of ‘no-go > go’ contrast in the non-learners is negative, suggesting a qualitative rather than just a quantitative difference in the pattern of activity in the IFG between learners and non-learners.

## Discussion

We report a striking asymmetry for instrumental learning, whereby participants were better at learning to emit a behavioral response in anticipation of reward, and better at withholding a response in anticipation of punishment. A computational analysis revealed that this corruption of instrumental action learning could be accounted for in terms of an influence of a Pavlovian learning system. The striatum and the SN/VTA tracked action values for both choices, but with opposite signs for go and no-go. This finding points to value representation being bound either to the regulation of vigor or, equivalently here, to the specification of the chosen behavior (go or no-go). Finally, selective recruitment of left SN/VTA and bilateral IFG was coupled to the emergence of successful instrumental control. The overall pattern of findings highlights a mandatory coupling between valence and action at the behavioral level that contrasts with a dominance of vigor control at the neurobiological level.

The data we report help refine the conception of Pavlovian influences over instrumental control as well as the architecture of instrumental decision-making itself. We note that our participants performed an apparently trivial task that entailed learning a simple relationship between four fractal images and a highly restricted behavior repertoire (go or a no-go choice). As the probability of reaping a reward or avoidance of a punishment was much higher for correct (0.8) than incorrect choices (0.2) one might expect rapid and fluent learning equivalent across all conditions. The striking finding that subjects, as a group, were impaired in this simple form of learning is testament to the strength and potential perniciousness of biases and asymmetries built into the architecture of decision-making. Furthermore, these effects persisted throughout a relatively lengthy learning period, and defeated optimizing instrumental learning mechanisms in a non-trivial fraction of our subjects.

Our computational modeling revealed that a key asymmetry in learning came from a coupling between valence and vigor. This coupling is central to classical Pavlovian to instrumental paradigms where the presentation of the Pavlovian stimulus modifies the vigor of instrumental responses in a valence dependent manner (Dickinson and Balleine, 2002; Huys et al., 2011; Talmi et al., 2008). That is, go was favored in conditions where there was a possibility of winning money and no-go when there was a possibility of losing, while the alternative mappings were difficult. This pattern of behavioral finding is consistent with a number of well-known results such as negative automaintenance (Dayan et al., 2006; Williams and Williams, 1969). Such deep embedding of strong biases within flexible instrumental mechanisms may serve to alleviate computational costs of learning. Conversely, such biases may also lie at the root of many anomalies of decision-making (Dayan et al., 2006; Guitart-Masip et al., 2010). Interestingly, the deleterious effects of punishment on go choices, but not the deleterious effects of reward on no-go choices, were also observed in our previous study (Guitart-Masip et al., 2011). In that study we used a similar paradigm but with the crucial difference being that participants were both instructed about contingencies and over-trained to reach high levels of accuracy on the go/no-go choices. One possibility suggested by this is that the certainty as to the correct choice may affect Pavlovian influences on action selection elicited by reward differently from those elicited by punishment.

Most previous human studies of learning have focused on two conditions that our subjects found straightforward: i.e., go to win and no-go to avoid losing (e.g. Cools et al., 2009; Frank et al., 2004; O'Doherty et al., 2004). A prevalent view is that dopamine projections to target structures, including the striatum (McClure et al., 2003; O'Doherty et al., 2003; Pessiglione et al., 2006), express reward prediction error signals (Bayer and Glimcher, 2005; Schultz et al., 1997) in the form of phasic bursts for positive prediction errors and dips below baseline for negative prediction errors (Bayer et al., 2007). The striatum then uses increases in dopamine to reinforce the direct pathway and generate go choices, while dips in dopamine reinforce the indirect pathway and generate no-go choices (Frank et al., 2004; Wickens et al., 2007). This functional architecture provides a plausible mechanism for instrumental learning of active responses through positive reinforcement and passive responses through punishment. Here, by passive we mean that they do not involve the generation of any overt behavioral responses. Crucially, in these straightforward conditions, instrumental and Pavlovian controllers prescribe the same action and are thus indistinguishable.

An instrumental system of this sort embodies an asymmetry since it provides no clear mechanism for learning to go in order to avoid losing or to no-go in order to win. One idea is that instrumental mechanisms treat conditions such as active avoidance by coding the removal of possible punishment as akin to a reward (Maia, 2010; Moutoussis et al., 2008; Mowrer, 1947). In support of this view, whereas dopamine deficits impair acquisition and maintenance of active avoidance behavior (Darvas et al., 2011; McCullough et al., 1993), learning about the prospect of punishment can occur even when dopamine is compromised (Beninger and Phillips, 1981). This implies that dopamine is required to learn a requirement for active responses to avoid punishment but another system learns about punishment itself. Serotonin has been suggested as being involved in coding for aspects of punishment or punishment prediction errors, although this is far from certain (Boureau and Dayan, 2011; Cools et al., 2011; Daw et al., 2002). If two stages are indeed involved in learning active avoidance, then this could also contribute to the observed behavioral asymmetry.

In line with the above view, our fMRI results showed that the striatum and the SN/VTA tracked action values for both go and no-go choices but that the relationship between value and brain activity was positive for go and negative for no-go. These results extended our recent observation that during anticipation, activity in striatum and lateral aspects of the SN/VTA complex reflect action requirements rather than state values (Guitart-Masip et al., 2011). It may be that both structures are part of an integral instrumental system that learn the value of available behavioral options, but where coding is relative to the control of vigor and approach. However, caution should be exercised in interpreting the lack of a conventional value signal in the striatum and the SN/VTA, as our experimental design did not allow us to search for such a signal in the current experiment.

Conversely, despite a clear effect of valence on action learning, we did not find any effect of valence on action value representations. This negative result does not imply that the observed behavioral asymmetry was not realized in the brain. It may arise as an example of the sort of malign valence-induced bias in learning that induces risk sensitivity (Denrell, 2007; March, 1996; Niv et al., 2002). That is, consider the no-go to win condition. If some participants happened to obtain reward for an early trial in which they performed a go response, they might continue performing go inflexibly, without sampling no-go. As both the Pavlovian and a value independent action bias favor the performance in the go to win condition, a reverse inflexible performance of an early rewarded no-go, is unlikely to manifest. Future experiments should be designed to dissociate state and action values. This would require that these two values are not highly correlated in the way there were in the current experiment. A possible strategy for future examination of this would be to include forced trials without choices, but only outcomes.

We also did not see any BOLD signals consistent with a prediction error at the time of the outcome. Since prediction errors are highly correlated with the reward term of the prediction error, and to ensure that a region is reporting a reward prediction error in a given task, it is necessary to separate the reward prediction in its two components, that is the reward and the value expectation (Behrens et al., 2008). In the current experiment, we followed this principle and only found a correlation between BOLD and the reward term at the time of the outcome. Similar results, in which prediction errors are not apparent, have previously been reported (Behrens et al., 2008; Li and Daw, 2011). Reconciling these results with those showing prediction errors (Daw and Doya, 2006) is an important task for the future. One possibility is that prediction errors in the striatum are only observed when prediction errors are of behavioral relevance for the instrumental task at hand (Klein-Flugge et al., 2011; Li and Daw, 2011). In the present task, the actual value of the stimuli was irrelevant for the instrumental task as instrumental choices could be informed with the reward component itself. In other context where participants may need to compare the relative value of different options, the full prediction error may be necessary for optimal instrumental performance as previously reported (e.g. Glascher et al., 2010; O'Doherty et al., 2004; Pessiglione et al., 2006; Schonberg et al., 2007).

The decrease in activity within the striatum and SN/VTA as no-go choice value increased does not fit the classical view of cortico-striatal circuits, in which reward promotes the direct (go) pathway and the punishment promotes the indirect (no-go) pathway (Frank et al., 2004; Hikida et al., 2010). Instead, a supplementary mechanism seems to be required. Indeed, we observed that during anticipation, before subjects actually performed a behavioral response (go or no-go), only subjects who learned the no-go to win condition recruited bilateral IFG in trials requiring inhibition of a go choice. Given the functional anatomy of IFG, it is interesting to speculate that those who learned the task did so by overcoming dominant go response tendencies, as for example when presented with a reward predicting fractal image that mandated a no-go choice. The same would be true for the no-go-to-avoid losing condition if as suggested by the model, participants must learn to overcome a value independence bias toward go choices in this task. Recruitment of IFG is systematically associated with an ability to stop a preponderant motor response (Aron and Poldrack, 2006; Robbins, 2007), or when there is a need to slow down in a decision task involving response conflict (Fleming et al., 2010).

Similarly, only participants who learned the appropriate choices in all conditions selectively recruited the left SN/VTA in trials requiring a go choice, suggesting that an inability to restrict such SN/VTA responses to go trials is related to a failure in learning task contingencies. We observed a similar pattern of activations in our previous study, in which the participants had such extensive training as to behave akin to learners during the second part of the experiment in the current task (Guitart-Masip et al., 2011). Within the limitation of fMRI studies of the SN/VTA (Duzel et al., 2009), this pattern is consistent with a suggestion that dopamine plays a role in action preparation and invigoration (Berridge and Robinson, 1998; Niv et al., 2007; Salamone et al., 2007), a role complementary to its established role in representing a reward prediction error.

Non-learners failed to acquire appropriate behavior in conditions where the choices prescribed by a Pavlovian controller were inappropriate. This echoes recent evidence regarding individual differences in decision-making, and most particularly a prominent distinction between sign-tracking and goal-tracking in rodents (Flagel et al., 2010; 2011). Just as for our non-learners, Pavlovian influences are dominant for sign-trackers. Interestingly, rats with lesions of the subthalamic nucleus showed increased sign-tracking behavior (Uslaner et al., 2008), and we note that the effects of the IFG in stopping go responses are mediated by the subthalamic nucleus (Aron and Poldrack, 2006). Furthermore, the STN is recruited by the IFG when a subject rejects a default choice (Fleming et al., 2010). This raises the possibility that the IFG, together with the subthalamic nucleus, complements an instrumental system by allowing it to overcome the vagaries of Pavlovian influences. An immediate question for future research would be how this complementary system is triggered if, as suggested in the current experiment, the IFG does not appear to track action values.

Our model captured a set of Pavlovian influences over behavior, with predictions of future reward being mandatorily associated with go active approach, and vigor; and predictions of future loss with a wider range of responses including no-go behavioral inhibition, and quiescence (Boureau and Dayan, 2011; Cools et al., 2011; Niv et al., 2007). Other possible substrates for these influences include the nucleus accumbens and the amygdala (Cardinal et al., 2002; Parkinson et al., 1999; Talmi et al., 2008) where dopamine plays a particularly important role in appetitive effects (Parkinson et al., 2002). On the other hand, serotonin is a prominent candidate for aversive effects (Dayan and Huys, 2009; Deakin and Graeff, 1991). Indeed, tryptophan depletion abolishes punishment induced inhibition, which is akin to the disadvantage we observed in the go to avoid losing condition (Crockett et al., 2009).

Our key finding was that during a simple form of instrumental learning, healthy human volunteers showed a striking interdependence of action and valence which exerted a corrupting effect on the course and outcome of learning. We captured this within a computational architecture that invoked distinct, albeit interacting, behavioral control systems, an instrumental and a Pavlovian system. We showed that the striatum and the SN/VTA tracked instrumental values in opposite ways for go and no-go choices, suggesting that these value representations are bound to a regulation of vigor. Thus, our data point to intriguing functional dissociations with these regions that enrich their putative roles beyond that associated with the generation and report of prediction errors.

The following are the supplementary data related to this article.

Fig. S1 Behavioral performance in learners and non-learners
