# Supplementary material for: Go and no-go learning in reward and punishment: Interactions between affect and effect
Source: Neuroimage. 2012 Aug 1;62-334(1):154–66. doi: 10.1016/j.neuroimage.2012.04.024 (PMC3387384; doi:10.1016/j.neuroimage.2012.04.024)

**SUPPLEMENTAL DATA**

**Figure S1: Behavioral performance in learners and non-learners**

Subjects were classified as learners if they performed more than 60% correct trials across the whole experiment and 80% correct in the second half of the experiment in every condition. By definition, learners performed better in every condition.

(A-D) Probability of a go response on every trial for each of the four conditions for learners (n=19; blue traces) and non-learners (n=11; purple traces).

(E) Mean fraction of correct responses in the four conditions. Blue bars depict learners, purple bars the non-learners. Green error bars depict the 95% confidence interval (CI) and the red error bars depict standard error of the mean (SEM). Both in learners and non-learners, a two way ANOVA with action (go/no go) and valence (win/lose) as repeated factors on fraction of correct responses revealed a significant action by valence interaction (learners F(1,18)=12.84, p=0.002; non-learners F(1,11)=36.55, p<0.001). A post hoc paired t-test in the learner group revealed a significant difference between the number of correct choices in the go to win condition and go to avoid losing condition (t(18)=4.04, p=0.001). On the other hand the number of correct choices in the no go to win condition and no go to avoid losing condition did not differ (t(18)=4.04, p=0.137), although subjects reached asymptote quicker in the no go to avoid losing than the no go to win condition. A three way ANOVA with time (6 time bins), action (go/no go), and valence (win/lose) as repeated factors revealed a significant action by valence by time effect F(5,90)=7.1, p<0.001, reflecting a greater number of correct choices in the second time bin in no go to avoid losing than in the no go to win condition (t(18)=2.35; p=0.031). Thus, the observed interaction between action and valence was a robust effect across the entire dataset, and was not driven by a subgroup of subjects that did not learn the task contingencies. However, the learners were able to overcome the difficulties posed by the asymmetric link between action and valence.

(F) Maximum a posteriori (MAP) parameter estimates of the best model for the learners (blue) and non-learners (purple). Green error bars depict the 95% confidence interval (CI) and the red error bars depict standard error of the mean (SEM). Comparisons were implemented by means of two sample t-tests: *P<0.005. This result shows that the main source of difference between learners and non-learners reflects a Pavlovian interference.


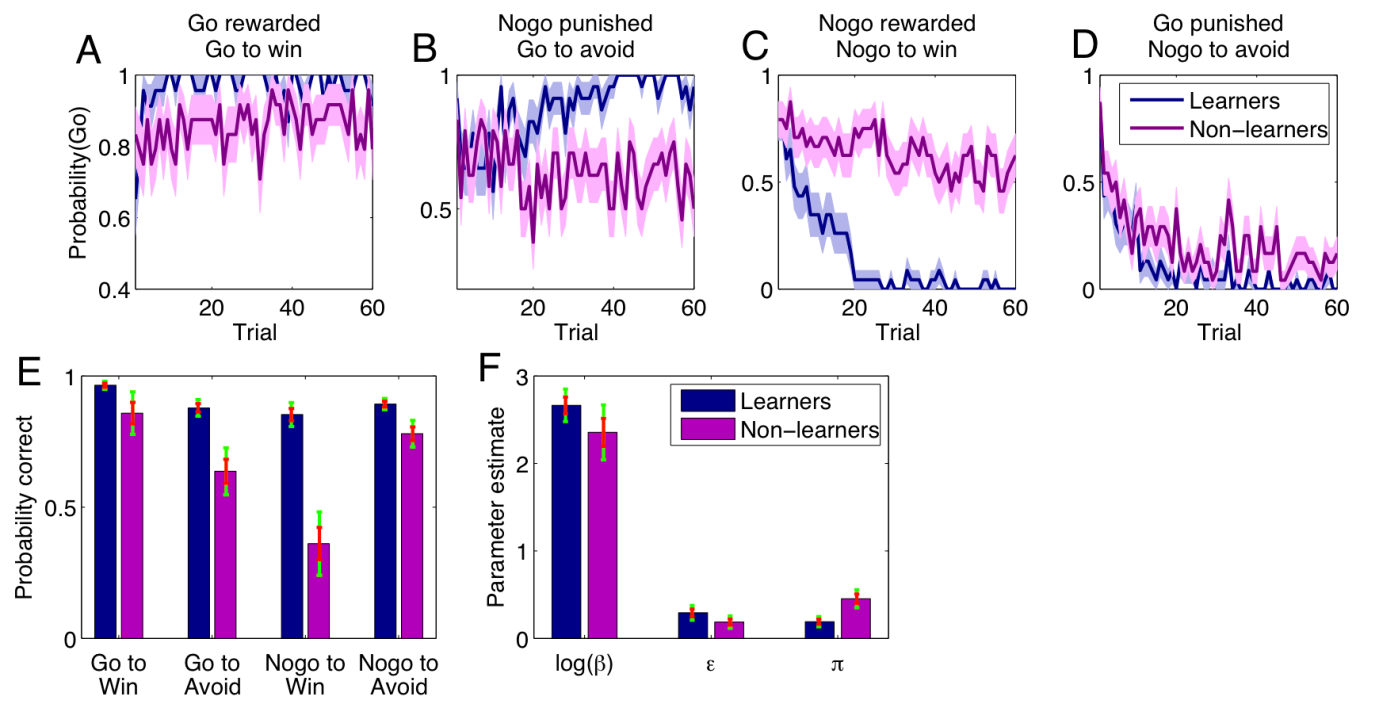

Supplement: Fig. S1 — Behavioral performance in learners and non-learners [file mmc1.doc]
